# The distinct clinical features of giant cell tumor of bone in pagetic and non-pagetic patients are associated with genetic, biochemical and histological differences

**DOI:** 10.18632/oncotarget.18670

**Published:** 2017-06-27

**Authors:** Giuseppina Divisato, Federica Scotto di Carlo, Laura Pazzaglia, Riccardo Rizzo, Domenico A. Coviello, Maria Serena Benassi, Piero Picci, Teresa Esposito, Fernando Gianfrancesco

**Affiliations:** ^1^ Institute of Genetics and Biophysics Adriano Buzzati-Traverso, National Research Council of Italy, Naples, Italy; ^2^ Department of Environmental, Biological and Pharmaceutical Sciences and Technologies (DiSTABiF), University of Campania Luigi Vanvitelli, Caserta, Italy; ^3^ Laboratory of Experimental Oncology, Rizzoli Orthopaedic Institute, Bologna, Italy; ^4^ Institute of Protein Biochemistry, National Research Council of Italy, Naples, Italy; ^5^ Laboratory of Human Genetics, Galliera Hospital, Genova, Italy; ^6^ IRCCS INM Neuromed, Pozzilli, Italy

**Keywords:** bone cancer, GCT, pagetic GCT, ZNF687, H3F3A

## Abstract

Giant Cell Tumor of Bone (GCT) is a tumor characterized by neoplastic mesenchymal stromal cells and a high number of osteoclast-like multinucleated giant cells. Rarely, GCT could arise in bones affected by Paget's disease of bone (GCT/PDB). Although it is already known that GCT/PDB and GCT show a different clinical profile regarding the age-onset and skeletal localization, our deep clinical comparison between the two GCT/PDB and GCT cohorts, permitted us to identify additional differences (e.g. focality, ALP serum levels, the 5-year survival rate and the familial recurrence), strongly suggesting a different molecular basis. Accordingly, driver somatic mutations in *H3F3A* and *IDH2* were described in GCT patients, while we recently identified a germline mutation in *ZNF687* as the genetic defect of GCT/PDB patients.

Here, we detected *H3F3A* mutations in our GCT cohort, confirming its molecular screening as the elected diagnostic tool, and then we excluded the *two-hit* in *H3F3A* and *IDH2* as the trigger event for the GCT/PDB development. Importantly, we also identified an alternative biochemical profile with GCT/PDB not exhibiting the up-regulation of the GCT marker *FGFR2IIIc*. Finally, our histological analysis also showed a different appearance of the two forms of the tumor, with GCT/PDB showing a higher number of osteoclast-like giant cells (twice), with an abnormal number of nuclei per cell, corroborating its different behaviour in terms of neoplastic properties.

We demonstrated that the distinct clinical features of pagetic and conventional GCT are associated with different genetic background, resulting in a specific biochemical and histological behaviour of the tumour.

## INTRODUCTION

Giant Cell Tumor of Bone (GCT), also referred to as osteoclastoma, is an osteolytic skeletal neoplasm characterized by a high population of multinucleated osteoclast-like (OCL-like) giant cells. Although the OCL-like population is a constant and prominent part of this tumor, it has been demonstrated that it is due to the uncontrolled proliferation of mesenchymal stromal cells, that mainly maintain the osteoclastogenesis instead of differentiating into osteoblasts [1–2].

Rarely, in less than 1% of cases, GCT can occur in patients affected by Paget's disease of bone (GCT/PDB), a focal disorder characterized by increased and disorganized bone remodelling, bone expansion, and abnormal bone structure [3–4]. In some instances, the diagnoses of PDB and GCT are made at the same time, whereas in most cases the diagnosis of PDB precedes the occurrence of GCT by about 12 years. To date, only 117 cases of GCT/PDB are reported in the literature [5]. Clinically, GCT and GCT/PDB show a different profile, regarding the frequency, the age-onset (20-40 yrs in GCT and >40 yrs in GCT/PDB) and the skeletal localization of the neoplasm [5–6]. Conventional GCT mainly affects the appendicular skeleton (distal femur, proximal tibia and knee in nearly 50% of cases) with a low likelihood of dissemination [6–7]; on the contrary, GCT/PDB affects the pagetic areas of the axial skeleton (skull, mandible and pelvis), with a preferential localization in the spine in PDB patients with multiple skeletal GCTs [5]. Conventional GCT standard treatments usually contemplate the surgery (curettage or resection) for the complete removal of the tumor [8]. However, GCT in some instances is a challenge to surgical treatments because of its proximity to the vital structures. To date, Denosumab is the first and only drug approved in the United States, Europe and Japan for treatment of unresectable GCT [9]. This full monoclonal antibody specifically acts targeting RANKL-positive stromal cells and interfering in the interaction with RANK-positive osteoclast-like giant cells, promoting the reduction of neoplastic stromal cells [10]. Moreover, the Denosumab effect also reflects on the reduction of osteoclast-like giant cell population, responsible for the osteolytic activity of both tumors [11–12]. However, data concerning the Denosumab treatment in pagetic GCT are few. Only two different studies reported one GCT/PDB case each, with an immediate tumor regression as response to Denosumab treatment, leading the authors to hypothesize that GCT/PDB should be treated with the same principles as that of conventional GCT [12–13].

Recently, driver somatic mutations in the *H3F3A* gene have been described as responsible for conventional GCT in the most cases (92%) [2]. Specifically, the p.Gly34Trp mutation, described in 48/53 conventional GCT cases and p.Gly34Leu mutation, in one case, were restricted to the mesenchymal population and not to the OCL-like giant cells, conferring the cancer property to the osteogenic stromal cell population [2]. Subsequently, p.Gly34Arg and p.Gly34Val mutations in the *H3F3A* gene were also identified in one and two cases of conventional GCT, respectively [14–15]. The cell-specific functions of *H3F3A* somatic mutations remain to be determined.

Another recent study showed that mutations in *IDH2* (p.Arg172Ser, p.Hys175Tyr) are responsible for conventional GCT in Asian patients without mutations in *H3F3A* [16]. In addition, *IDH1* mutation (p.Arg132Pro) contributes to the bone tumors onset by dysregulating the differentiation of mesenchymal stromal cells [17]. *IDH1/2* mutations result in an overproduction of 2-hydroxyglutarate, a metabolite inhibiting H3K27 and K36 demethylases [16]. The pathogenic mechanism responsible for GCT development is still poorly understood and even more prominent is the gap existing about the GCT/PDB pathogenesis. However, Singh *et al*. recently observed a very high expression of Fibroblast Growth Factor Receptor 2-IIIc isoform (*FGFR2-IIIC*) and twist family bHLH transcription factor 1 (*TWIST1*) in GCT stromal cells, suggesting that they play an essential role in these cells [18]. In addition, two other targets frequently found expressed in GCT tumor tissues are the glycoprotein of the extracellular matrix tenascin C (TNC) and the Epidermal Growth Factor Receptor (EGFR), described associated with GCT tumor progression. However, even less is known about the biochemical alteration in pagetic GCT [19–20]. From a genetic point of view, we identified the responsible mutation (p.Pro937Arg) in patients with GCT/PDB in the *ZNF687* gene that encodes a component of the Z3 complex involved in interpreting the histone code for chromatin remodelling for transcription [21–22]. Therefore, in this study we first fully elucidated the genetic basis of these two forms of the tumor and then investigated their putative biochemical pathways and histological appearance.

## RESULTS

### The atypical clinical presentation of pagetic giant cell tumor

To further investigate the clinical differences between these two forms of the tumor, we compared clinical features of our cohort of 100 conventional GCT patients with those of 117 GCT/PDB cases collected from the literature, that we recently revised [5].

As expected, GCT/PDB patients developed the tumor at a mean age significantly higher (62.5±11.7 yrs) than conventional GCT patients (33.2±8.4 yrs) (Table 1).

**Table 1:** Clinical characteristics of conventional and pagetic GCT patients

	GCT n=100	GCT/PDB n=117*
Age at onset of GCT	33.2±8.4	62.5±11.7
Single GCT (%)	99	75
Multifocal GCT (%)	1	25
GCT involving axial skeleton (%)	6	75
GCT involving appendicular skeleton (%)	94	25
5-year survival rate (%)	>95	<50
High ALP serum levels at GCT onset (%)	rare	98.6
Family history (%)	none	35

* GCT/PDB data derived from Rendina et al. 2015

Moreover, the clinical characterization of the patients underlined the main involvement of the axial skeleton in GCT/PDB (75%), in contrast to the involvement of the appendicular skeleton (94%) observed in conventional GCT. The higher severity of GCT/PDB phenotype was corroborated by the multifocal behaviour of the tumor, developing multiple neoplasms in more than 25% of patients. In contrast, only one patient of our cohort of conventional GCT showed the involvement of two skeletal sites (femur and tibia) confirming the quite total monofocal form of this tumor. The dramatic picture for GCT/PDB was also reinforced by its 5-year survival rate (less than 50% of the patients survived) as well as the high levels of ALP serum at GCT onset detected in 98.6% of patients. Finally, from a genetic point of view, none of our conventional GCT patients showed other affected relatives, while we described a familial recurrence for GCT/PDB in 35% of patients [5].

### Different genetic signatures for conventional and pagetic giant cell tumor

Driver mutations have been identified in the *H3F3A* gene (p.Gly34Trp, p.Gly34Leu, p.Gly34Val, p.Gly34Arg) as responsible for conventional GCT in more than 90% of Caucasian patients [2, 14]. On the contrary, somatic mutations in the *IDH2* gene were described as the genetic defect in 80% of Asian GCT patients [16].

**Figure 1:**
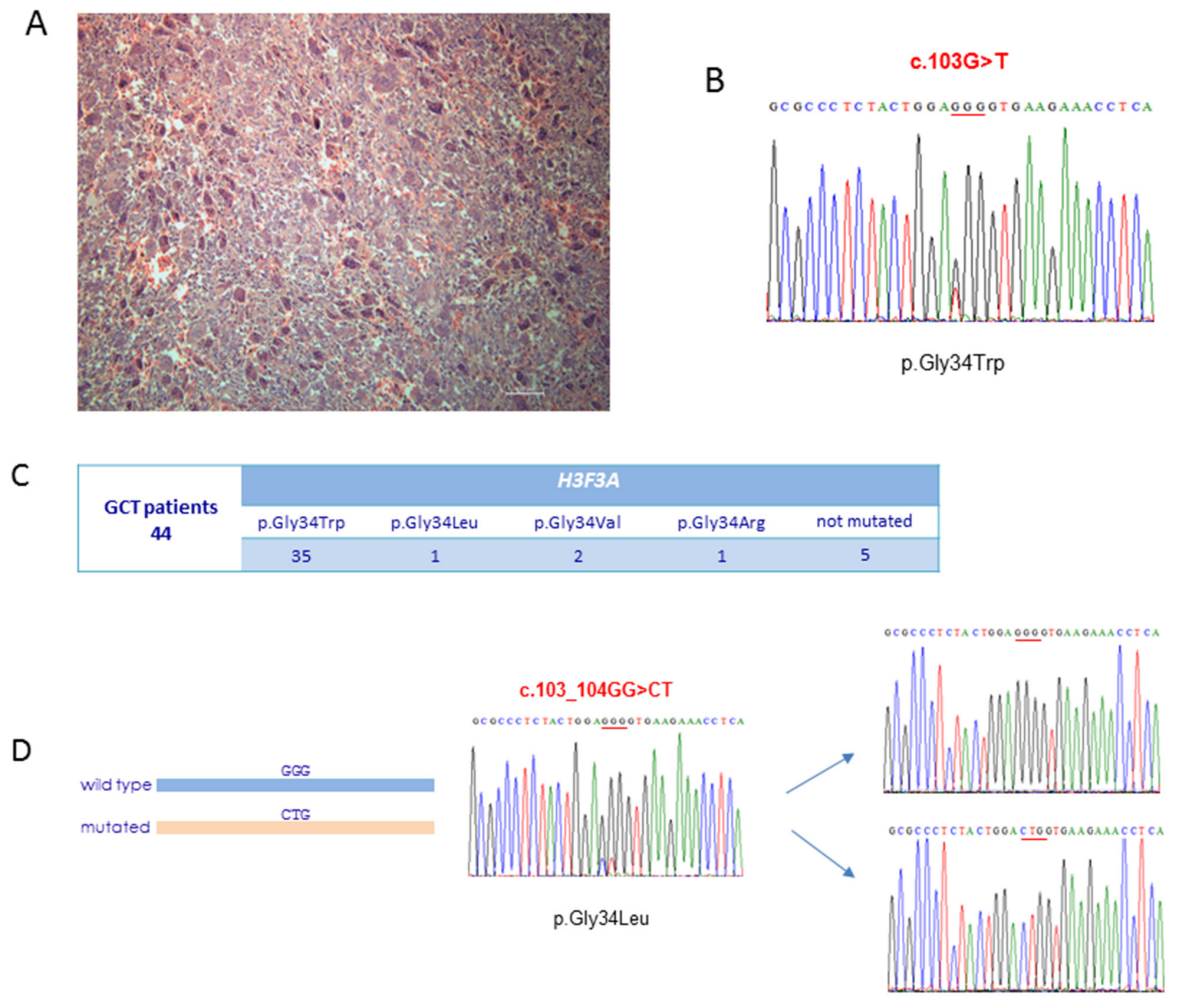
*H3F3A* molecular screening **(A)** Haematoxylin and eosin staining of conventional GCT biopsy used for DNA extraction and carrying p.Gly34Trp somatic mutation in *H3F3A*. **(B)** The DNA sequence of a segment flanking p.Gly34Trp somatic mutation in *H3F3A* from a conventional GCT patient. **(C)** Output of the *H3F3A* targeted sequencing in our cohort of 44 conventional GCT patients. **(D)** Allele specific sequencing of cloned DNA fragment containing p.Gly34Leu somatic mutation in *H3F3A* resulting from a substitution of two adjacent nucleotides.

To elucidate the genetic basis of our cohort, we performed *H3F3A* targeted sequencing on DNA extracted from tumour biopsies of a subset of 44 GCT patients (Figure 1A). Our somatic analysis revealed *H3F3A* mutations in 39 out of 44 cases (89%), confirming *H3F3A* molecular screening as the elected diagnostic tool for Caucasian patients with conventional GCT [15]. In particular, we detected the most common mutation p.Gly34Trp in 35 patients and rarely p.Gly34Arg (1 patient), p.Gly34Leu (1 patient), p.Gly34Val (2 patients) (Figure 1B and 1C). As the p.Gly34Leu mutation is the result of the substitution affecting two adjacent nucleotides (c.103_104GG>CT), in order to reveal if these two changes were located on the same allele, we cloned the DNA fragment containing this mutation and performed the allele specific sequencing, confirming their cis-configuration (Figure 1D).

To verify if the co-occurrence of *H3F3A* and *IDH2* somatic mutations was associated with poorer outcome in GCT patients, we performed *IDH2* molecular screening in patients carrying *H3F3A* somatic mutations and also in those (5 out of 44) negative for mutations. We did not detect any alteration, confirming the exclusivity of *H3F3A* mutations in Caucasian patients as well as *IDH2* mutations in Asian population, as already described [16].

Recently, we reported a founder germline mutation (p.Pro937Arg) in the *ZNF687* gene as responsible for the Giant Cell Tumor arising on Paget's disease of bone [21]. Since we identified the same mutation in PDB patients without neoplastic transformation and also considering that *H3F3A* and *IDH2* mutations occur at somatic level, we performed molecular analysis of both genes on tumor tissues derived from 5 GCT/PDB patients, carrying the germline mutation in *ZNF687*. None of the examined biopsies harboured a mutation neither in these candidate genes nor in the entire coding regions of PDB-related genes (*SQSTM1* and *ZNF687*). Nevertheless, the presence of unidentified somatic mutations for GCT/PDB elsewhere in the genome could justify a *two-hit* as trigger event for GCT development in bones previously affected by PDB. Finally, to exclude the co-occurrence of *ZNF687* and *H3F3A* mutations, we also performed genetic analysis of *ZNF687* in all conventional GCT cases carrying the somatic mutations in *H3F3A*, not identifying any mutation in the *ZNF687* gene. All together, these data confirmed two different genetic signatures for these two forms of the tumor.

### Absence of expression of the GCT marker FGFR2IIIc in GCT/PDB

**Figure 2:**
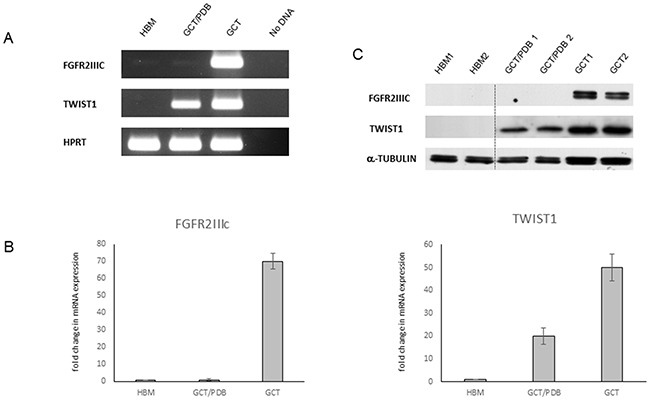
Differential expression of FGFR2IIIc and TWIST1 in GCT and GCT/PDB **(A)** RT-PCR and **(B)** qRT-PCR in healthy bone marrow (HBM), GCT/PDB and GCT showing *FGFR2IIIc* and *TWIST1* expression profile (fold of expression). The results are expressed as the fold change compared to healthy bone marrow samples, using the ddCT method. **(C)** FGFR2IIIc and TWIST1 protein levels are showed.

Considering the genetic differences between GCT and GCT/PDB, we decided to investigate if also an alternative biochemical behaviour of the two tumor forms occurred. In particular, we analysed the endogenous expression in 5 GCT/PDB tumor tissues of two markers, as *FGFR2IIIc* and *TWIST1*, already described as up-regulated in GCT [18]. Through qRT-PCR and RT-PCR assays, we confirmed the upregulation of *FGFR2IIIc* in 5 GCT tumor tissues but surprisingly, we observed a very low-to-almost-undetectable expression level for this marker on RNA derived from GCT/PDB tumor tissues (Figure 2A and 2B). Consistently, western blot analysis showed the total absence of FGFR2IIIc in GCT/PDB and its remarkable up-regulation in GCT (Figure 2C).

Moreover, these analyses also highlighted a mild upregulation of *TWIST1* in GCT compared to GCT/PDB and confirmed the absence of expression of this marker in healthy bone marrow, as already described (Figure 2A-2C) [23].

**Figure 3:**
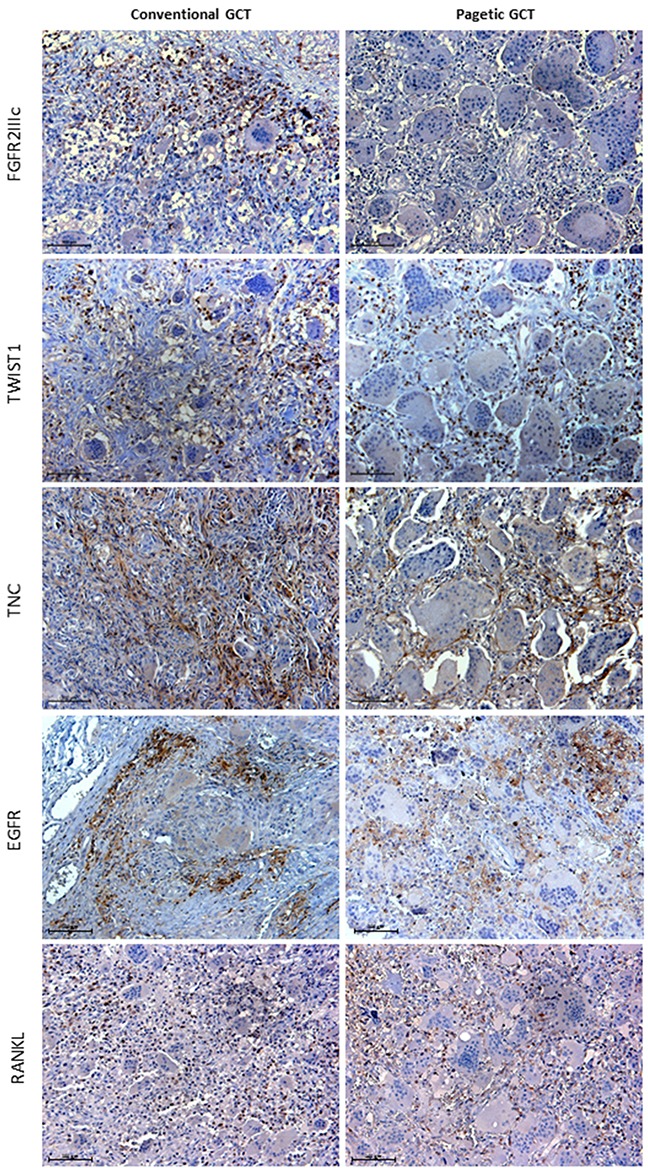
Histological comparison of the GCT markers between conventional and pagetic GCT FGFR2IIIc immunoreactivity was detected in mesenchymal GCT cells interspersed among the negative OCL-like giant cells, while it was absent in GCT/PDB tumor tissue. TWIST1 immunostaining was observed in the nuclei of mesenchymal stromal cells and in their cytoplasm (moderate signal) in both tumors. TNC detection showed reticulate-fibrillar pattern in GCT and GCT/PDB tumor tissues mainly referred to the spindle mononuclear cells. EGFR localised in cell membranes of the spindle shaped mononuclear cells. RANKL showed similar distribution in mesenchymal stromal cells in both tumors.

Subsequently, immunohistochemistry assay provided information about the cell population expressing these two markers. As expected, our analysis revealed that the spindle mononuclear GCT cells strongly expressed FGFR2IIIc, whereas it resulted undetectable in the GCT/PDB tissue (Figure 3). On the contrary, in both tumor forms we detected a strong TWIST1 expression in the nuclei of mesenchymal lineage cells and a moderate signal in their cytoplasm (Figure 3).

In order to verify whether other already described markers also showed differences between GCT and GCT/PDB, we investigated the immunoreactivity of Tenascin C (TNC) and Epidermal Growth Factor Receptor (EGFR) as well as Receptor Activator of Nuclear factor Kappa-B Ligand (RANKL), known to play a central role in GCTs (Figure 3).

Collectively, we did not find any difference between GCT and GCT/PDB tumor tissues. In fact, TNC showed a distribution referred to the matrix and the mesenchymal mononuclear cells with reticulate-fibrillar pattern, as already described (Figure 3) [19]. EGFR resulted mainly referred to the cell membrane of the spindle-shaped mononuclear cells as reported by *Balla et al. 2011* (Figure 3) [20]. Finally, we detected RANKL-positive stromal cells in both tumors equally distributed among the negative OCL-like giant cells (Figure 3).

### Different histological appearance for conventional and pagetic giant cell tumor

**Figure 4:**
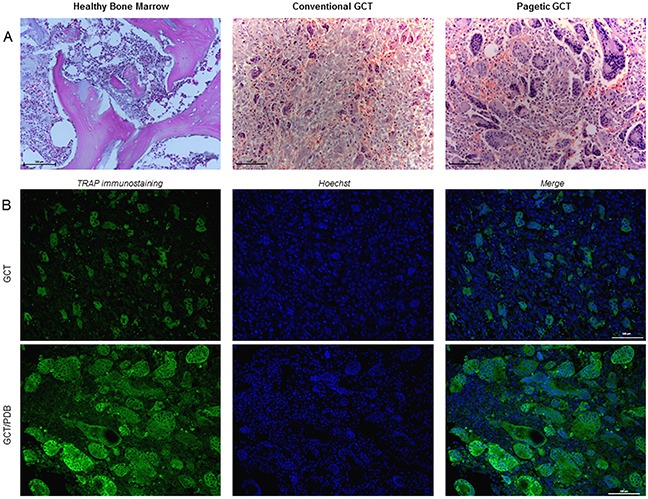
The different histological appearance of GCT and GCT/PDB tumor tissues **(A)** Haematoxylin and Eosin staining on GCT (*H3F3A*, p.Gly34Trp) and GCT/PDB (*ZNF687*, p.Pro937Arg) tumor biopsies showed a different histological appearance for GCT/PDB, resulting in a higher number of OCL-like multinucleated giant cells. **(B)** TRAP-positive OCL-like giant cell count revealed a double number of cells in GCT/PDB compared to conventional GCT.

**Figure 5:**
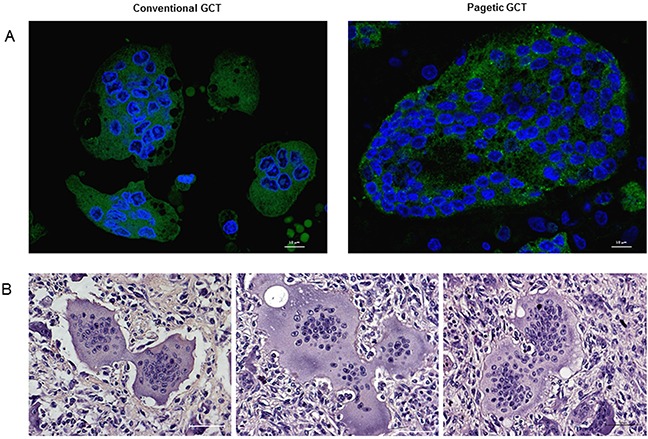
OCL-like giant cells characterization through cytomorphometric analysis **(A)** Confocal counting revealed bigger OCL-like giant cells with an increased number of nuclei compared to conventional GCT, also reflecting a different nucleus-cytoplasm ratio. **(B)** Haematoxylin and Eosin staining highlighted cell-cell contacts between multinucleated giant cells in both forms of tumors.

Since previous IHC experiments led us to suspect a different histological appearance between these two forms of the tumor, we decided to perform *ad hoc* Haematoxylin and Eosin staining on GCT and GCT/PDB tumor biopsies, bearing the most frequent *H3F3A* (p.Gly34Trp) and *ZNF687* (p.Pro937Arg) mutations, respectively. This analysis confirmed that GCT/PDB was characterized by a higher number of OCL-like multinucleated giant cells (Figure 4A). To obtain a more specific demonstration of countable and measurable giant cells, we performed immunofluorescence-based staining for the osteoclastic marker Tartrate-resistant Acidic Phosphatase (TRAP), evaluating different parameters as the number of cells, number of nuclei, cell size, nuclei size and nucleus-cytoplasm ratio. As expected, we found a significant high number of TRAP-positive multinucleated OCL-like giant cells in GCT/PDB that resulted about double as compared to conventional GCT (Figure 4B). With a similar approach we extended our cytomorphometric analysis to the nuclei number, in order to detect any difference between conventional and pagetic OCL-like giant cells. We determined the nuclei number of OCL-like giant cells, counting a mean number of 150 nuclei (maximum 168) per cell in GCT/PDB tumor biopsies, while we counted a mean number of 25 nuclei (maximum 48 nuclei) per cell in conventional GCT, demonstrating that pagetic OCL-like giant cells resulted increased in number of cells and number of nuclei (Figure 5A). Pagetic OCL-like giant cells also showed a 4-fold increase in the size as compared to conventional OCL-like giant cell size (451±50 μm^2^ *vs* 103±19 μm^2^), while we did not find significant differences in the nuclei size (35±5 μm^2^ *vs* 40±9 μm^2^), that was similar in both cases (Figure 5A). Finally, in pagetic OCL-like giant cells we also observed a different nucleus-cytoplasm ratio (0.6±0.05) compared to conventional OCL-like giant cells (0.4±0.09).

## DISCUSSION

GCT is a locally aggressive lesion consisting of mononuclear stromal cells and multinucleated giant cells that exhibit osteoclastic activity [6]. Typically, this osteolytic bone neoplasm could appear in its conventional form or related to other bone diseases, as Paget's disease of bone, showing a different clinical profile about the age-onset of the tumor and its skeletal localization [1, 5]. Here, by comparing the clinical features of our cohort of conventional GCT patients with those of 117 GCT/PDB cases collected from the literature, we noted a more aggressive phenotype for GCT/PDB, since they showed a multifocal behaviour of the tumor (the patients developed multiple neoplasms) and a lower 5-year survival rate (less than 50% of the patients survived).

On this basis, one of our objectives was to determine whether these distinct clinical features of conventional and pagetic GCT are associated with a different genetic, biochemical and histological background. This purpose is justified by the alternative genetic profile that we recently identified in GCT/PDB individuals, harbouring the founder germline mutation (p.Pro937Arg) in the *ZNF687* gene, compared to driver somatic mutations in the *H3F3A* and *IDH2* genes in the conventional form of GCT [21, 2, 16].

Therefore, we first confirmed the *H3F3A* genetic screening as the elected diagnostic tool for conventional GCT, identifying mutations in the 89% of our patients, in concordance with those recently reported [2, 15]. Then, we excluded the *two-hit* model in the *H3F3A* gene as trigger event for the GCT transformation of the pagetic bone performing somatic molecular analysis on 5 GCT/PDB tumor tissues carrying the *ZNF687* germline mutation. Additional studies (exome sequencing) are necessary to reveal if somatic mutations are located elsewhere in the genome. However, all together these results provide a molecular tool (*ZNF687* and *H3F3A* screening) to obtain a differential diagnosis bypassing the clinical overlap between these two forms of the same tumor that could appear in some instances (e.g. unclear age of onset and/or undefined skeletal localization).

To date, the molecular mechanism through which these two genes cause GCT is unknown and further studies are needed to elucidate if *ZNF687* and *H3F3A* are in the same biochemical pathway. Both genes are involved in the epigenetic mechanisms, considering that ZNF687 interacts with the histone reader ZMYND8 and that *H3F3A* encodes the histone variant H3.3. However, our results suggest a totally or partially different biochemical pathway for GCT/PDB as demonstrated by the absence of the upregulation of the GCT marker FGFR2IIIc. A possible scenario could contemplate two different tumorigenic insults, both culminating in the formation of the multinucleated giant cells. In addition, the similar expression pattern of other markers, known to be expressed in conventional GCT (e.g. TWIST1, TNC, EGFR, RANKL), strongly supported the evidence that the sole difference between these two tumors only regarded FGFR2IIIc.

Finally, we also demonstrated that the different molecular profile is responsible for a different histological appearance of the tumor with GCT/PDB showing a higher number of bigger TRAP-positive OCL-like giant cells with a higher number of nuclei.

Although the mechanism of multinucleation in giant cell tumor is not completely understood, the cell fusion is supposed to be the most probable mechanism. It has been hypothesized that multinucleated giant cells result from fusion of the proliferating mononuclear cells, through tumor-induced cell fusion [24–25]. In addition, in our analysis, we also observed cell-cell contacts between multinucleated giant cells in both forms of tumor, suggesting that the big size as well as the multinucleation could result from the fusion of osteoclast-like giant cells (Figure 5B). However, functional evidence is missing and further studies are necessary.

In conclusion, our study demonstrates that the distinct clinical features of conventional and pagetic GCT are associated with different genetic background, resulting in a specific biochemical behaviour and histological appearance of the tumour.

## MATERIALS AND METHODS

### Ethics statement

A cohort of 100 GCT and 5 GCT/PDB patients recruited from Orthopaedic Rizzoli Institute (IOR) was included in this study. The samples were collected at the time of surgery and one section was immediately snap-frozen and stored at – 80°C, while the other section was formalin fixed, and the clinical diagnosis was histologically confirmed by Haematoxylin and Eosin (H&E) staining. Written patient informed consent was obtained individually. The clinical profile of 117 GCT/PDB, that we systematically reviewed from the literature, was used to define the clinical differences between GCT/PDB and our GCT cases [5].

### *H3F3A*, *IDH2* and *ZNF687* molecular analysis

The targeted somatic sequencing was performed on a subset of 44 GCT and 5 GCT/PDB tumor tissues. The isolation of high-pure genomic DNA was obtained using High Pure PCR Template Preparation Kit (Roche), according to the manufacturer's protocol. *H3F3A* (ENSG00000163041.9), *IDH2* (ENSG00000182054.9), *ZNF687* (ENSG00000143373.17) and *SQSTM1* (ENSG00000161011.19) were amplified by polymerase chain reaction (PCR), followed by automated DNA sequencing. PCR amplifications were carried out by using Taq DNA polymerase (1 U; Fermentas, Glen Burnie, MD, USA). The samples were ExoSap-digested (Amersham) and sequenced using the Big Dye Terminator Ready Reaction Kit (Applied Biosystems, Foster City, CA, USA) on the ABI Prism 3710 Genetic Analyser (Applied Biosystems).

### Allele-specific sequencing

The molecular cloning of *H3F3A* DNA sequence containing c.103_104GG>CT mutation was obtained amplifying the fragment using Phusion High-Fidelity DNA Polymerase (ThermoFisher). The resultant fragment was subcloned into pJET1.2/blunt cloning vector (ThermoFisher) and transformed to DH5α E. coli bacteria. Allele-specific sequencing was performed using Sanger method.

### RNA isolation and RT-PCR

Frozen GCT and GCT/PDB tumor biopsies were disrupted and homogenised using the Tissue Lyser LT (Qiagen) for 5 min at 50 Hz in TRIZOL Reagent (Sigma) according to the manufacturer's protocol. RNA concentration and purity were confirmed by absorbance measurement, using Nanodrop 2000c (Thermo Scientific) followed by agarose gel electrophoresis. One microgram of total RNA was reverse transcribed with the RevertAid RT kit (Thermo Scientific). RT-PCRs and qRT-PCRs were performed by using SYBR Select Master Mix for CFX (Applied Biosystems) on Bio-Rad CFX Connect Real-Time System instrument. The expression of the hypoxanthine-guanine phosphoribosyltransferase (*HPRT*) was used as internal control. The experiments were carried out in triplicate.

### Protein extraction and Western blotting analysis

Total protein extraction from GCT and GCT/PDB tumor tissues was performed by using RIPA Buffer (Tris-HCl pH 7.5 50mM, NaCl 150 mM, DTT 1 mM, sodium fluoride 50 mM, sodium deoxycholate 0.5%, SDS 0.1%, NP-40 1%, phenylmethanesulfonyl fluoride 0.1 mM, sodium vanadate 0.1 mM) with 1X proteinase inhibitor mixture (Sigma Aldrich). Tissues disruption and homogenization was performed using Tissue Lyser LT (Qiagen), for 5 min at 50 Hz. Total proteins were quantified using the Bradford method. Protein samples were separated by SDS-PAGE, blotted onto PVDF membrane (Millipore). Western blotting was performed using rabbit polyclonal anti-Bek (c-17): sc-122 (Santa Cruz), rabbit polyclonal TWIST1-specific 25465-1-AP (Proteintech) and mouse monoclonal anti-α-Tubulin T6074 (Sigma Aldrich) antibodies.

### Histological analyses

Formalin-fixed paraffin-embedded (FFPE) GCT and GCT/PDB tumor slices were stained with Haematoxylin and Eosin (H&E), according to the standard procedure. Briefly, the sections were deparaffinized in changes of xylene and rehydrated in decreasing concentrations of ethanol. GCT and GCT/PDB tumor slices were incubated with haematoxylin solution (Sigma Aldrich) for ten minutes and after rinsing in distilled water, they were stained with eosin for ten minutes. The sections were dehydrated in ethyl solution of gradient concentrations, hyalinized in xylene and mounted by neutral balsam. Optical microscope (Leica DM6000) was used to observe the stained biopsies. For immunohistochemistry assays, antigen retrieval was obtained boiling the specimens in a mixture of 0,1 M Tris-base and 0,01M ethylenediamine tetraacetic acid (EDTA) (pH 9.0; Tris/EDTA). The sections were immunostained with the following primary antibodies: rabbit polyclonal anti-Bek (c-17): sc-122 (Santa Cruz), rabbit polyclonal TWIST1-specific 25465-1-AP (Proteintech), mouse monoclonal anti-Tenascin C ab6393 (Abcam), mouse monoclonal EGFR (A-10): sc-373746 (Santa Cruz Biotechnology) and rabbit polyclonal RANKL ab9957 (Abcam). Positive controls were included for each antibody and negative controls were prepared omitting the primary antibody. The slices were incubated with the biotinylated secondary antibodies (VECTOR Laboratories). Diaminobenzidine (DAB) was used for the development.

### TRAP immunofluorescence

TRAP immunofluorescence assay was performed on FFPE sections, deparaffinised and rehydrated following the procedure described above. For antigen detection the sections were incubated with mouse monoclonal antibody Tartrate Resistant Acid Phosphatase (26E5) MA5-12387 (Thermo Fisher Scientific) followed by the incubation with the secondary antibody Alexa Fluor 488 goat anti-mouse IgG. The nuclei were counterstained with Hoechst33342 (Lonza). Finally, they were analysed using a Nikon's A1R Confocal laser microscope. NIS Elements software was used to perform OCL-like cells and nuclei counts on GCT and GCT/PDB tumor biopsies, randomly selected. TRAP-positive cells with more than 3 nuclei per cell were considered multinucleated OCL-like giant cells. The counting was performed using three 20x microphotographs derived from each sample and calculated as the mean count in the 3 microphotographs of several biopsies carrying the same mutation.
